# Question answering system using Q & A site corpus Query expansion and answer candidate evaluation

**DOI:** 10.1186/2193-1801-2-396

**Published:** 2013-08-22

**Authors:** Kanako Komiya, Yuji Abe, Hajime Morita, Yoshiyuki Kotani

**Affiliations:** Institute of Engineering, Tokyo University of Agriculture and Technology, 2-24-16 Nakacho-Kotanei, Tokyo, 184-8588 Japan; Tokyo Institute of Technology, 4259 Nagatsuta, Midori-ku, Yokohama, Kanagawa, 226-8503 Japan

## Abstract

Question Answering (QA) is a task of answering natural language questions with adequate sentences. This paper proposes two methods to improve the performance of the QA system using a Q&A site corpus. The first method is for the relevant document retrieval module. We proposed modification of measure of mutual information for the query expansion; we calculate it between two words in each question and a word in its answer in the Q&A site corpus not to choose the words that are not suitable.

The second method is for the candidate answer evaluation module. We proposed to evaluate candidate answers using the two measures together, i.e., the Web relevance score and the translation probability. The experiments were carried out using a Japanese Q&A site corpus. They revealed that the first proposed method was significantly better than the original method when their accuracies and MRR (Mean Reciprocal Rank) were compared and the second method was significantly better than the original methods when their MRR were compared.

## Introduction

Question Answering (QA) is a task of answering questions written in natural language with adequate sentences, which consists of the following four modules (Soricut and Brill 2006).

1. Question analysis
2. Relevant document retrieval
3. Candidate answer extraction
4. Candidate answer evaluation

When a question written in natural language is input into the system, the system carries out keyword extraction in the question analysis module. Then the system retrieves relevant documents using the keywords that were obtained in the last module in the relevant document retrieval module. After that, the system extracts candidate answers in the candidate answer extraction module. The size of the candidate answers varies according to their question types, e.g., a phrase or a sentence. A sentence or a paragraph will be the candidate answer when the QA is non-factoid. Finally, the system estimates the qualities of the candidate answers that were obtained in the candidate answer extraction module in the candidate answer evaluation module.

This paper proposes two methods to improve the performance of the QA system using a Q&A site corpus. The first method is for the relevant document retrieval module. We proposed modification of measure of mutual information for the query expansion. The query expansion is an approach to extend query words by adding new words that are not included in each question to improve the qualities of the relevant documents to be retrieved. In previous work, words to be added are chosen based on mutual information between a word of each question and a word of its answer in the Q&A site corpus (Berger et al. 2000). We calculated it between two words in each question and a word in its answer in the Q&A site corpus not to choose the words that are not suitable for the query expansion.

The second method is for the candidate answer evaluation module. The QA system estimates the qualities of candidate answers that were obtained by the document retrieval in this module. This module is important because it directly affects system’s outputs. There are two cues to estimate candidate answers, i.e., 1) the topic relevance, which evaluates association between each candidate answer and its question in terms of its content, and 2) the writing style, which evaluates how the writing style of each candidate answer corresponds to its question type. In this paper, we propose to evaluate candidate answers using the Web relevance score (Ishioroshi et al. 2009) and the translation probability (Soricut and Brill 2006) together.

We will show that our proposed methods improved each module by the experiments using a Japanese Q&A site corpus.

This paper is organized as follows. Section ‘Related work’ reviews related work on QA. Sections ‘Query expansion using mutual information’ and ‘Query expansion using two words in a question’ explain how words for query expansion were determined in the relevant document retrieval module in the previous work (Berger et al. 2000) and the first proposed method for the module, respectively. Sections ‘Candidate answer evaluation’ and ‘Candidate answer evaluation with web relevance score and translation probability’ describe how candidate answers were evaluated in the candidate answer module in the previous work (Ishioroshi et al. 2009 and Soricut and Brill 2006) and the second proposed method for the module. Section ‘Experiments’ explains the experimental settings. We present the results in Section ‘Results’ and discuss them in Section ‘Discussion’. Finally, we conclude the paper in Section ‘Conclusion’.

## Related work

Question Answering (QA), which involves answering questions written in natural language with adequate sentences, has been studied intensively in recent years within or outside the area of natural language processing. The QA systems within the area are sometimes called as open domain question answering systems because they are not domain specific (Ishioroshi et al. 2009).

Types of questions that are treated by the QA systems can be categorized into two kinds, i.e., factoid and non-factoid. Questions of the former type ask the names of people or places, or the amounts of stuffs, e.g., “How tall is Mt. Fuji?”. On the other hand, questions of the latter type ask definitions, reasons, or methods, e.g., “What are ES cells?”. Our system treats the both types of questions in this paper.

We proposed two methods to improve the performance of the QA system; the first method is for the query expansion of the relevant document retrieval module and the second method is for the candidate answer evaluation module. For the query expansion, Saggion and Gaizauskas (2004) proposed to obtain words for the query expansion using relevance feedback from the Web. They regarded words that appeared frequently in documents retrieved for each question query as the new words for the query expansion. Mori et al. (2007) and Derczynski et al. (2008) used tf-idf and Lin et al. (2010) used Okapi-BM25 for the criteria instead of the term frequency of Saggion and Gaizauskas (2004). Lao et al. (2008) proposed to obtain the synonyms of words in each question using bootstrap method and to use them for the query expansion. Saggion and Gaizauskas (2004) also used synonyms but obtained them from a dictionary. Liu et al. (2010) obtained them from Wikipedia. Finally, Berger et al. (2000) proposed to learn what kind of words tend to appear in answers when some words appeared in questions using a Q&A site corpus and to use words that frequently appear for the query expansion. We improved one of the approaches suggested by Berger et al. (2000) in this paper.

For the query expansion, some researchers such as Higashinaka and Isozaki (2007,2008) and Isozaki and Higashinaka (2008) reported that the performance of the system improved when the question types were classified into classes such as “how-questions” and “why-questions” in advance. However, Ishioroshi et al. (2009) and Soricut and Brill (2006) developed a QA system without classification of the question types. Ishioroshi et al. (2009) estimated the topic relevance by relevance feedback from the Web.

Soricut and Brill (2006) and Berger et al. (2000) treated QA task as translation and succeeded in evaluating the topic relevance and the writing style simultaneously. We also improved them by combining their methods together without classification of the question types.

## Query expansion using mutual information

Berger et al. (2000) proposed to learn what kind of words tend to appear in answers when some words appeared in questions using a Q&A site corpus and to use words that frequently appear for the query expansion. In their work, mutual information was used to measure the degree of relevance between a word in each question and a word in its answer. The formula of mutual information is as follows: 1

where *W*_*q*_ and *W*_*a*_ represent binary random variables that show whether a word *w*_*q*_ appear in each question and whether a word *w*_*a*_ appear in its answer, respectively. 23

The more *w*_*q*_ and *w*_*a*_ co-occur in a corpus, the grater their mutual information becomes.

Berger et al. (2000) chose a word from its answer for every words in each question. It was the word that maximized mutual information between the question word and the answer word itself. After this, {a word in a question → a word in an answer} denotes the query expansion using this method.

This method works effectively when the training and test corpora are domain specific^a^. However, it sometimes causes semantic drift when corpora are large and not domain specific. For example, when the question was “What are the connections between softbank and yahoo?”, it gave us the following results: {softbank → hawks}^b^ and {yahoo → mail}. *Hawks* and *mail* are relevant with *softbank* and *yahoo*, respectively, but they should not be used for the query expansion because they are no relevance with the original question.

## Query expansion using two words in a question

In order to alleviate the semantic drift, we propose to use mutual information based on two words in each question and a word in its answer. The new equation of mutual information is as follows: 4

It represents the degree of co-occurrence between two words in a question and a word in its answer. The more *w*_*q*1_, *w*_*q*2_, and *w*_*a*_ co-occur in a corpus, the grater their mutual information becomes like equ. (1). For example, when the question was “What are the connections between softbank and yahoo?”, it gave us the following results: {softbank and yahoo → subsidiary}.

## Candidate answer evaluation

The second proposed method is for the candidate answer evaluation. As mentioned above, the topic relevance and the writing style are used to estimate candidate answers. We introduced two existing methods for the module. First method is the work proposed by Ishioroshi et al. (2009), which estimated the topic relevance by relevance feedback from the Web. They regarded words that frequently appeared in documents retrieved for each question query as relevant words. Therefore, candidate answers that contain many relevant words were regarded better in terms of the topic relevance.

The relevance words are obtained as follows:

1. Make a keyword class K that contains content words (i.e., nouns, verbs, and adverbs) in each question
2. Choose three words from K in all combinations and search the Web by them
3. Obtain at most 100 Web snippets, i.e., summaries of the Web documents that were obtained by a Web search engine, for each query

Each content word *w*_*j*_ in these snippets is treated as a relevant word for the question. The relevance degree of the relevant word, i.e., *T*(*w*_*j*_) is defined by the following equation: 5

where *i* represents a index of a query (i.e., triple of content words), *n* denotes the number of snippets obtained from *i*_*th*_ query, *freq*(*w*_*j*_,*i*) denotes the number of snippets that contain word *w*_*j*_ that were obtained from *i*_*th*_ query. Candidate answer evaluation score in terms of the topic relevance, i.e., *Web*_*relevance*(*Q*,*A*) is defined as the sum of the relevant degrees of the relevant words contained in each candidate answer as follows: 6

where *Q* represents a question, *A* represents its candidate answer, *l* denotes the number of words in the candidate answer, and *w*_*j*_ denotes each word in the candidate answer.

Finally, Ishioroshi et al. (2009) evaluated candidate answers using the following score that took into consideration the topic relevance and the writing style as well: 7

where *l* denotes the number of types of word *w*_*i*,*j*_ in a sentence *S*_*i*_, *m* represents the number of types of writing feature *b*_*i*,*k*_ in *S*_*i*_, *length*(*S*_*i*_) means the number of the characters of *S*_*i*_, *χ*^2^ denotes the score of each writing style, and *γ* represents the weighting parameter. As for *χ*^2^, a chi-square value is calculated between the answers that include the writing feature *b*_*i*,*k*_ and the top N answers retrieved for the question query.

The second method is the work by Soricut and Brill (2006), which treated QA task as translation. They succeeded in evaluating the topic relevance and the writing style simultaneously. In their method, each question and its answer are regarded as the source and target sentences, respectively. For translation, word-by-word translation probabilities are learned using a Q&A site corpus. When a question is input into the system, this system calculates the translation probabilities from the question into their candidate answers. Then the candidate answers are evaluated using their probabilities. They used the IBM-Model1 (Brown et al. 1993) as a translation model, which is simple but showed efficacy in many tasks. The answer evaluation is formulated as follows using IBM-Model1 (as in Berger et al. (2000)) : 89

where *A*^⋆^ represents the most adequate candidate answer, *Q*(=*q*_1_,*q*_2_,…,*q*_*m*_) and *A*(=*a*_1_,*a*_2_,…,*a*_*l*_) each represents a question and its candidate answer, *m* and *l* each denotes the number of words in the question and its candidate answer^c^, *P*(*q*|*a*) represents the translation probability from a word *a* in an answer to a word *q* in a question, *c*(*a*_*i*_|*a*) are the relative counts of the answer words, *P*(*A*) denotes generation probability of the candidate answer *A*, and *ε* is a probability of generating a question whose length is *m* from the candidate answer.

We can have the equation (10) by assuming that *c*(*a*_*i*_|*a*) is 1/*l* like Brown et al. (1993). 10

In equation (10), there is a problem where the less the number of words in a candidate answer becomes, the more its translation probability increases because the value of the coefficient increases as *l* decreases. Therefore, we neglected the coefficient and got equation (11) instead of equation (10). 11

## Candidate answer evaluation with web relevance score and translation probability

When evaluating the topic relevance, the method using the translation probability proposed by Soricut and Brill (2006) can flexibly capture synonyms. This is because the translation probabilities are learned from the massive examples of a Q&A site corpus beforehand. However, it is unable to capture the co-occurrence information of several words in a question because it only utilizes word-to-word translation probabilities. By contrast, the Web relevance score proposed by Ishioroshi et al. (2009) can capture the co-occurrence information but cannot capture the synonyms because the Web documents dynamically obtained are small. Thus, it seems that the answer evaluation method using these methods simultaneously would be able to achieve the greater performance.

### New answer evaluation formula

Equation (12) is the new formula of the answer evaluation that uses the Web relevance score and the translation probability: 1213

where  represents the probability that should be maximized in the equation (8) (the score by Soricut and Brill (2006)), *Web*_*relevance*(*Q*,*A*) denotes the score using Web relevance score (the score by Ishioroshi et al. (2009)), and *γ* represents the weighting parameter. The equation (12) is equivalent to the translation probability when *γ*=0 whereas it is the same as the Web relevance score when *γ*=1.

## Experiments

Two kinds of the experiment were carried out using a Japanese Q&A site corpus, i.e., the 100 questions of “NTCIR-ACLIA2” (Mitamura et al. 2010), as the test questions. The “Yahoo! Chiebukuro” data were used as examples of a Q&A site corpus for calculation of mutual information and for training of the translation probability. The “Yahoo! Chiebukuro” data is distributed to researchers from the National Institute of Informatics based on a contract with the Yahoo Japan Corporation (National Institute of Informatics 2009). “Yahoo! Chiebukuro” is the largest knowledge retrieval service in Japan, and the Yahoo Japan Corporation has been providing this service since April 2004. Their aim is to connect people who want to question and those who want to answer, and the sharing of wisdom and knowledge among the participants. The National Institute of Informatics provides data consisting of 3.11 million questions and 13.47 million answers (total text size of 1.6 billion characters) submitted between April 2004 and October 2005 out of about 10 million questions and about 35 million answers currently stored. The 100 questions of “NTCIR-ACLIA2” is included in NTCIR-8 ACLIA test collection (National Institute of Informatics 2012). This test collection includes 100 Japanese topics of Mainichi News Paper, which consists of 377,941 documents between years 2004 and 2005. It can be used for experiments of Complex Question Answering.

Morphological analysis was only carried out in the question analysis module although some works such as (Oda and Akiba 2009) and (Mizuno et al. 2007) classified question types there. ChaSen (Kyoto University and NTT 2013) was used as a morphological analyzer and the Yahoo! API (Yahoo Japan Corporation 2013) was used as a search engine. A candidate answer is always a sentence since we did not classify the question type. Web documents were retrieved for a query of all the question’s content words with or without query expansion and were used as the source of the candidate answers. Thus, we did not have tagged answers.

### Experiments of query expansion

The experiments were carried out as follows for each question. First, words were chosen from answers of a Q&A corpus as candidates for the query expansion. Here, each single word was chosen for every combination of two words in question for the system of the proposed method. By contrast, each word was chosen for every word in question for the system of the original method. Next, the top three words at most are chosen in the order of mutual information as the words to be added for the query expansion. Finally, the candidate answers were retrieved and evaluated.

**Figure 1:**
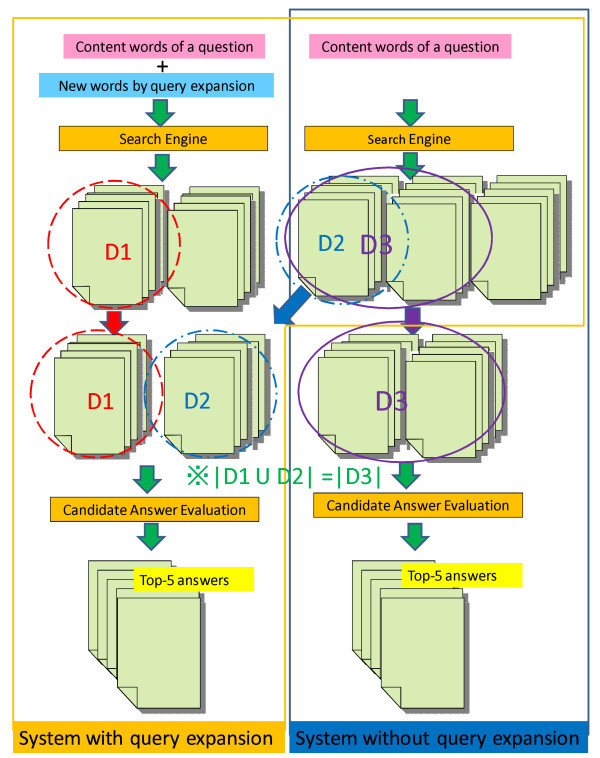
**Outlines of Web document retrieval and candidate answer evaluation with and without query expansion.** This figure shows the outlines of the Web document retrieval and the candidate answer evaluation with and without query expansion. *D*1∪*D*2 and *D*3 each represents the candidate answers with and without query expansion. The number of the documents of *D*1∪*D*2 was equalized to that of *D*3 for the fair comparison.

Figure 1 shows the outlines of the Web document retrieval and the candidate answer evaluation of the systems with and without query expansion. The documents were retrieved from the Web two times for the system with the query expansion: using all content words and using all content words and all new words for query expansion. The candidate answers were collected from the two sets of document retrieved by the system. On the other hand, the documents were retrieved from the Web only once for the system without query expansion: using all content words. The candidate answers were collected from them. *D*1 is a subset of documents retrieved by a query with the query expansion and *D*2 and *D*3 are subsets of documents retrieved by a query without query expansion in Figure 1. *D*1∪*D*2 and *D*3 each represents the candidate answers with and without query expansion. The number of the documents of *D*1∪*D*2 was equalized to that of *D*3 for the fair comparison; we set it to 80 documents. The score proposed by Ishioroshi et al. (2009) (the score of equ. (7)) was used for the candidate answer evaluation. The weighting parameter *γ* is set to 0.5. Unigrams were used as the feature of the writing style.

### Experiments of candidate answer evaluation

GIZA++ (Casacuberta and Vidal 2007), which is the implementation of IBM-Model1, was used as a learning tool for the translation probability. The number of iterations of EM-algorithm was set to five times. The examples of a Q&A site corpus whose question or answer contains more than 60 words were preliminarily cut off because they negatively affected the learning of word alignment; they contained too many words. Moreover, the examples of a Q&A site corpus whose number of the words in the question is more than five times as many as that in the answer were cut off and vice versa for the same reason. As a result, 1,092,144 examples in the “Yahoo! Chiebukuro” data were used as the training data of GIZA++.

Fifty Web documents retrieved for a query without query expansion were chosen as the candidate answers and were evaluated by the proposed or original formula of the answer evaluation.

The bigrams normalized by the number of words were used for *P*(*A*).

## Results

Each candidate answer retrieved from Web documents was evaluated in the answer evaluation module and the QA system output the top-5 answers. The outputs of the system were checked manually. The top-5 accuracies and the MRR (Mean Reciprocal Rank) of the QA system were evaluated. The answer the system output is correct if it is in the top-5 answers when the top-5 accuracy is calculated. The top-5 accuracy is formulated as follows: 14

where *answered*_*question* is the number of the question where the system output the correct answer in the top-5 answers. MRR is formulated as follows: 15

where *rank*(*i*) represents the best rank of the correct answer of the *i*th question. MRR takes into consideration the rank of the output whereas the top-5 accuracy does not.

### Results of query expansion

Table 1 shows the top-5 accuracies and MRR of the experiments of the query expansion. The original method in the table represents the method of Berger et al. (2000), where words to be added are chosen based on mutual information between a word from each question and another word from its answer. This table shows the system with the proposed method outperformed the system without query expansion and the system with the method of Berger et al. (2000). It also showed that the system with the method of Berger et al. (2000) is inferior to the system without query expansion. We think this is because the large corpus we used caused the semantic drift. Thus, we think the method of Berger et al. (2000) is unsuitable for the open-domain QA.

**Table 1:** **Results of experiments of query expansion**

	Without query	Original method	Proposed method
	expansion		
Accuracy	0.420	0.400	0.450
MRR	0.262	0.233	0.273

This table summarizes the top-5 accuracies and MRR of the systems for the experiments of the query expansion. Original method in the table represents the method proposed by Berger et al. (2000), where the words to be added are chosen based on mutual information between a word from a question and another word in its answer. This table indicates that the system with the proposed method outperformed the two systems: the system without query expansion and the system with the method proposed by Berger et al. (2000).

On the other hand, the proposed method can choose words to be added for the query expansion without the semantic drift, because it considers the co-occurrence of not only one word but also two words from each question and another word from an answer. The difference between the original method and the proposed method was significant though the difference between the system without query expansion and the proposed method was not, according to a Wilcoxon signed-rank test. The significance level was 0.05.

### Results of candidate answer evaluation

**Figure 2:**
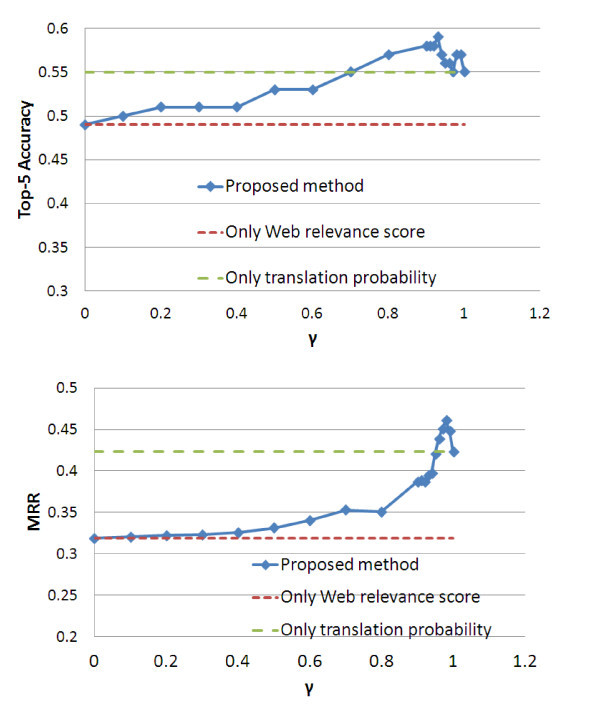
**Performance of proposed method.** This figure shows the top-5 accuracies and MRR of the experiments of the candidate answer evaluation when the value of *γ* changed from 0 to 1. The top-5 accuracy was maximized to 0.59 when *γ* = 0.93 and the MRR was maximized to 0.461 when *γ* =0.98.

Figure 2 shows the top-5 accuracies and MRR of the experiments of the candidate answer evaluation when the value of *γ* changed from 0 to 1. Table 2 lists the performances of the original methods and the proposed method. Table 2 shows that the top-5 accuracy was maximized to 0.59 when *γ* = 0.93. In addition, the MRR was maximized to 0.461 when *γ* =0.98. As for the MRR, the proposed method was significantly better than the original methods according to a Wilcoxon signed-rank test. The significance level was 0.05.

**Table 2:** **Results of experiments of answer candidate evaluation**

	Top-5 accuracy	MRR
Only Web relevanve (*γ*=1)	0.55	0.423
Only translation probability (*γ*=0)	0.49	0.318
Proposed method (*γ*=0.93)	0.59	0.395
Proposed method (*γ*=0.98)	0.57	0.461

This table summarizes the top-5 accuracies and MRR of the systems for the experiments of the candidate answer evaluation. As for MRR, the proposed method was significantly better than the original methods according to a Wilcoxon signed-rank test.

## Discussion

We will show examples of the results and discuss them in this section.

### Query expansion

The examples A, B, and C show the examples from the experimental results.

**Example A** Some examples where the new found word was the answer to the question could be found.

**Question**

“(What are the connections between softbank and yahoo?)”

**Query expansion via the original method**

{ (softbank) → (hawks)} and { (yahoo) → (mail)}

**Query expansion via the proposed method**

{  (softbank) and  (yahoo) → (subsidiary)}

According to Example A, we can see that the direct answer to the question was selected as a word for the query expansion via the proposed method. Even if the word *subsidiary* is not the direct answer, it is suitable for the query expansion because it has close connections with *softbank* and *hawk*.

**Example B** Some examples where the new found word was a clue to the question were also found.

**Question**

“(Let me know events related to the peace plan of India and Pakistan?)”

**Query expansion via the original method**

(India) → (curry)} and { (Pakistan) → (Islamic)}

**Query expansion via the proposed method**

{ (India) and  (Pakistan) → (Kashmir)}

Example B is a QA where the system cannot answer in a word; it is a non-factoid question. *Kashmir* is an important word because it is area that is close to India and Pakistan. On the other hand, *curry*, the word that is irrelevant to the question, was chosen via the original method. These words would cause the semantic drift, which sometimes makes it difficult to find documents that are relevant to the question. These words were frequently chosen via the original method, which decreased the performance of the system. We think that these cases did not happen in the experiments by Berger et al. (2000) because they used relatively small and domain specific corpora,

On the contrary, the proposed method where the system chooses the words that maximize mutual information between two words from a question and one word from its answer chose these words less frequently than the original method. It enabled better document retrieval for the QA that is not domain specific.

**Example C** These were some examples where the new found words were irrelevant to the question.

**Question**

“(Why did the price of crude increase in year 2004?)”

**Query expansion via the original method**

{ (increase) → (oil)} and { (year, age) → (marriage)}

**Query expansion via the proposed method**

{ (increase) and  (year, age) → (player or athlete)}

{ (does) and  (year, age) → (marriage)}

Words that are irrelevant to the question were chosen for the query expansion even via the proposed method in Example C. There were many cases like them when general words were used for the calculation of mutual information. Therefore, we think that the words to calculate mutual information should be carefully selected in the future.

### Candidate answer evaluation

#### Web relevance score

We will discuss about how scores of the topic relevance from the Web contributed the results. Examples D and E have examples of the Web relevance score for factoid and non-factoid questions, respectively. Web relevance scores of the words in answers are shown in brackets. Those of the words in questions were omitted.

**Example D** The relevant words could be obtained via the Web relevance score for some factoid questions.

**Question**

“(Where does the Olympic hold in 2008?)”

**Example of answer**

“” “(Beijing)”

**The relevant words**

, Beijing (0.67), , place (0.29), , convention (0.29), 11 (0.24), 2011 (0.24), 12 (0.23), , times (0.23), , Japan (0.23), , Tokyo (0.22), , represent (0.21), , game (0.2), 20 (0.2), , summer (0.19), , winter (0.19), , China (0.19) ⋯

Direct answers could be obtained when the question was factoid as shown in Example D. We could particularly obtain *Beijing*, which was related to both *2008* and *Olympic*, although we could hardly obtain these words via the method using only translation probability that can only take into consideration one word at a time. 

**Example E** The relevant words could be obtained via the Web relevance score for some factoid questions.

**Question**

“(What are ES cells?)”

**Example of answer**

“(The research team of Center of Developmental Biology, Institute of Physical and Chemical Research in Kobe, whose leader is Yoshiki Sasai, succeeded in largely culturing embryo-stem cells (ES cells), which have ability to be any types of cells in various tissues in body, and effectively changing them into cerebral cells.)”

**The relevant words**

, sex (0.29), , research (0.26), , stem (0.24), , human (0.23), , embryo (0.2), , differentiation (0.18), , mouse, mice (0.18), , remodeling, regeneration (0.16), , tissue (0.15), , medical (0.13), , like (0.13), , body (0.13), , science (0.12), , culture (0.11), iPS (0.11), ⋯

Direct answers to the question could not be obtained when the question was non-factoid. However, the words that are related to the question could be obtained. The suitable answers that include the relevant words could be also obtained as shown in Example E. However, words that frequently appear in many documents could not be distinguished from those that co-occur with content words in the question using mutual information. Thus, we think that the selection of these words using IDF will be able to be tried in the future.

#### Translation probability

We will discuss about how the translation probability contributed the results.

Table 3 has examples of the top-5 words that maximize *P*(*q*|*a*), which is the translation probability from a word *a* in an answer to a word *q* in a question when *a* is given. The English words and the numbers in brackets are the English translations and the translation probabilities, respectively. For example, when “” (medical care) was given as a word in an answer, it tended to be translated into “” (medical care), “” (hospital), “” (fare), “” (medical admission), and “” (operation) in its question. This indicates that “” (medical care) tends to appear in the answer when these words appear in its question. The functions of Japanese words are shown when the English words are written in upper case.

**Table 3:** **Examples of translation probability**

Index	Given word	1st	2nd	3rd	4th	5th
(1)		(.064)	(.057)	(.037)	(.026)	(.024)
	Medical care	Medical care	Hospital	Fare	Admission	Operation
(2)		(.097)	(.032)	(.021)	(.016)	(.016)
	Lawsuit	Lawsuit	Judgment	Sue over	Advocate	Right
(3)		(.081)	(.034)	(.025)	(.023)	(.022)
	Salt water	Salt water	Water	Tastes salty	Method	Shell
(4)		(.033)	(.020)	(.019)	(.015)	(.015)
	Landform	Landform	Yokohama	Times	Typhoon	Geography
(5)		(.115)	(.0373)	(.024)	(.024)	(.021)
	Channel	Channel	Island	World	Takeshima	Tohoku
(6)		(.249)	(.060)	(.031)	(.028)	(.023)
	Arrestment	Arrestment	PASSIVE	Get caught	Do	PAST
(7)		(.132)	? (.031)	(.030)	(.029)	(.026)
	Cell	Cell	?	PREDICATION	Why	Human
(8)		(.146)	(.060)	(.031)	(.031)	(.030)
	Information	Information	Of	PREDICATION	Do	AGENT
(9)		(.134)	(.065)	(.040)	(.039)	?(.038)
	Technology	Technology	Of	TOPIC MARKER	PREDICATION	?
(10)		(.298)	(.098)	(.039)	(.025)	?(.021)
	President	President	America	Bush	Of	?
(11)		(.222)	(.138)	(.047)	(.040)	(.030)
	Prime minister	Prime minister	Koizumi	Minister	Mr.	Prime minister
(12)		(.191)	?(.170)	(.143)	(.079)	(.045)
	Shepherd’s-purse	Seven herbs	?	As for	Of	QUESTION
(13)		?(.064)	(.063)	(.052)	(.049)	(.046)
	Because	?	Of	PREDICATION	TOPIC MARKER	QUESTION
(14)		(.073)	(.058)	? (.056)	(.050)	(.046)
	Because	Of	PREDICATION	?	QUESTION	AGENT
(15)		(.088)	(.061)	? (.055)	(.052)	(.048)
	For	Of	PREDICATION	?	QUESTION	For
(16)		(0.17)	(0.04)	(0.04)	(0.04)	(0.03)
	Reason	Reason	Why	AGENT	Of	QUESTION

This table has examples of the top-5 words that maximize *P*(*q*|*a*), which is the translation probability from a word *a* in an answer to a word *q* in a question when *a* is given. The English words and the numbers in brackets are the English translations and the translation probabilities, respectively. The functions of Japanese words are shown when the English words are written in upper case.

Table 3 firstly shows words in answers are likely to be translated into themselves in their questions. This indicates that words in questions tend to appear in their answers. Next, the table shows words in answers are likely to be translated into their relevant words and synonyms as shown in the case where (1) “” (medical admission) and “” (operation) for “” (medical care), and (11) “” (prime minister) for “” (prime minister) are listed in the table. This indicates that relevant words and synonyms of words in question tend to appear in their answers.

The properties of the relevant words and the synonyms that were obtained using the translation probability are different from those obtained from 100 Web documents because they were from approximately one million examples of a Q&A site corpus. Therefore, we think that the performance of the QA system improved because the Web relevance score and the translation probability complemented one another.

We expected that (13) “” (because), (14) “” (because, from), and (15) “” (because, for) were likely to co-occur with “” (why) or “” (why), which often appeared in questions, because they often appeared in answers of QA, but they did not. We think that this is because the particles like “” (because, from) and “” (because, for) are ambiguous. Soricut and Brill (2006), who used an English Q&A corpus for learning, reported that “because” tended to be translated into “why”. We think that the method worked well because the English word “because” was less ambiguous than Japanese words like “” (because, from) and “” (because, for).

However, (16) “” (reason), which is also likely to appear in answers to why-type questions, could be leaned as the word that tended to be translated into “” (why). This indicates that learning with the translation probability could be able to partially evaluate the writing style.

In addition, the words that appeared few times tended to be learned not correctly. For example, (12) “” (shepherd’s-purse) were hardly translated into relevant words because it appeared only twice in the Q&A site corpus. Moreover, some unsuitable words were chosen because the translation probabilities only depended on the Q&A site corpus. The “Yahoo! Chiebukuro” data are examples of Q&A site submitted from April 1st 2004 to October 31th 2005. Therefore, “” (Koizumi), who was the prime minister at that time, and “Bush”, who was the president of USA at that time, were chosen as the words likely to be translated from “” (prime minister) and “” (president), respectively.

## Conclusion

Question Answering (QA) is a task of answering natural language questions with adequate sentences. It includes the relevant document retrieval and candidate answer evaluation modules. This paper proposed two methods to improve the performance of the QA system using a Q&A site corpus. The first method is for the query expansion in the relevant document retrieval module. We proposed modification of measure of mutual information for the query expansion; we calculate it between two words in each question and a word in its answer in the Q&A site corpus not to choose the words that are not suitable. The second method is for the candidate answer evaluation module. We proposed the method to evaluate candidate answers using existing two methods, i.e., the Web relevance score and the translation probability. We showed that the proposed method evaluated the candidate answers more effectively than the original methods. The experiments were carried out using a Japanese Q&A site corpus. They revealed that the first method was significantly better than the original method when the accuracies and MRR were compared. They also showed that the second method was significantly better than the original methods when the MRR were compared.

## Endnotes

^a^ Berger et al. (2000) used Usenet FAQ documents and customer service call-center dialogues from a large retail company.

^b^ We got this word because we had a baseball team named softbank hawks in Japan.

^c^*P*(*q*_*j*_|*a*_*i*_) was summed from 1 to *l*+1 because each question word had exactly one connection to either a single answer word or empty.
